# Preservative Effects of *Gracilaria* sp. Extract in Maintaining the Quality and Shelf Life of Refrigerated Pangas (*Pangasius hypophthalmus*) Fillets

**DOI:** 10.1002/fsn3.70683

**Published:** 2025-07-20

**Authors:** Md Apon Dulal, Rokshana Islam, Shoumik Ahmed, Chunhong Yuan, A K M Azad Shah

**Affiliations:** ^1^ Department of Fisheries Technology Gazipur Agricultural University Gazipur Bangladesh; ^2^ Faculty of Agriculture Iwate University Morioka Japan; ^3^ Agri‐Innovation Center Iwate University Morioka Japan; ^4^ Institute of Food Safety and Processing Gazipur Agricultural University Gazipur Bangladesh

**Keywords:** Gracilaria‐based coating, pangas fillet quality, refrigeration storage, sensory attributes, shelf life

## Abstract

Quality deterioration and alterations in the sensory parameters of the pangas fillet in refrigerated conditions are the relative causes of enzymatic and bacteriological effects. This experiment was conducted to analyze the impacts of *Gracilaria* sp. extracts (GE) on the storability of refrigerated pangas fillets for 15 days. The fillets were dipped into various concentrations (1%, 2%, and 3%) of GE. The control (without GE) and GE‐treated fillets were kept at 4°C ± 1°C to determine chemical (pH, total volatile basic‐nitrogen (TVB‐N), free fatty acid (FFA), peroxide value (PV), and thiobarbituric acid reactive substances (TBARS)), bacteriological, and sensory attributes. Results showed that the pH, FFA, PV, TBARS, and TVB‐N values of 2% GE‐treated fillets had 6.92%, 1.57% oleic acid, 18.50 meq O_2_/kg lipid, 0.84 mg MDA/kg, and 22.78 mg N/100 g, respectively, at Day 12th, which were significantly (*p* < 0.05) lowered compared to other GE‐treated fillets. The maximum aerobic plate count was 7.15, 7.62, 7.45, and 7.11 log CFU/g at the 9th, 12th, 15th, and 12th days for control, 1%, 2%, and 3% GE‐treated fillets, respectively. The sensory attributes indicated that 2% GE‐treated fillets had better sensory characteristics, followed by 3% GE, 1% GE, and control fillets. The results stated that the control fillets got spoiled after 6th day, whereas 2% GE‐treated fillets were consumable up to the 12th day of refrigerated storage. Based on the results, 2% GE enhanced the shelf life by 6 days more than the control fillets under refrigerated conditions and could be a treasured natural source of fish preservatives.

## Graphical abstract

This study intended to investigate the preservative effects of *Gracilaria* sp. extracts on the quality and shelf life of pangas fillets during refrigerated storage. Results revealed that 2% *Gracilaria* sp. extracts had a significant impact on the physio‐chemical, microbial, and sensory attributes of fish fillets and enhanced the shelf life by 6 days more than the control fillets under refrigerated conditions.

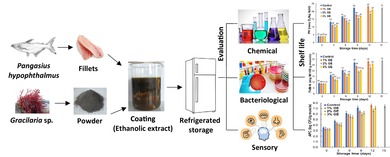

## 1 Introduction

Bangladesh is famous for its aquaculture production throughout the world and has achieved a remarkable output of 4.915 million MT in FY 2022–23 (Department of Fisheries [DoF] 2022). Pangas ( *Pangasius hypophthalmus* ) is a highly productive aquaculture species in Bangladesh and has a great contribution (8.21%) to the total fish production. It has gained popularity in Bangladesh due to its nutritional value, adaptability, and low price. Poor people also like this species for containing polyunsaturated fatty acids and many health benefits (Sokamtea et al. 2020). In this industrialization era, people have limited time to prepare food items and therefore, they often prefer fish fillets over whole fish. However, the fillets of this species are very perishable because of high water content, endogenous enzymes, and the presence of polyunsaturated fatty acids. In addition, various biochemical reactions (protein and lipid degradation) and microbial actions produce many non‐nitrogenous and volatile compounds (primary hydroperoxides, free fatty acids, aldehydes, and ketones) (Tran et al. 2021). The production of these compounds leads to organoleptic changes (color, odor, texture, etc.) in fish and reduces the shelf life in any conditions. To cope with this situation, the storage of fish by traditional technologies including refrigeration, chilling, and freezing conditions is not sufficient. Antioxidants are compounds that are capable of reducing lipid oxidation and prolonging the shelf life of fish. Therefore, various unauthorized, illegal, and health‐issue chemical antioxidants such as BHA, BHT, sorbate, benzoic acid, sodium benzoate, nitrite, sulfites, etc., are used in fish preservation (Rathod et al. 2021). The excessive application of these synthetic antioxidants to obstruct lipid oxidation and microbial spoilage leads to various health problems such as oxidative stress, DNA damage, cytotoxicity, and carcinogenicity in humans (Xu et al. 2021). Moreover, these substances exhibit long‐term toxicity that is the consequence of being endocrine disruptors (Lahreche et al. 2022). For this reason, it is important to use natural antioxidants as alternatives to synthetic ones, particularly because they are generally recognized as safe (Pateiro et al. 2018). Natural antioxidants found in seaweed extracts enhance the shelf life of fish and have attracted significant interest from researchers, primarily due to their antioxidant and antibacterial compounds (Shahrier et al. 2023; Deepitha et al. 2021).

Rhodophyta is the largest group of seaweeds, highly available in the intertidal zone of Bangladesh's coast. It contains various antioxidants, antimicrobials, and antioxidative color pigments such as chlorophyll *a* and chlorophyll *d*, carotenoids, and phycobiliproteins that exert many pharmaceutical and preservative properties (Carpena et al. 2023). *Gracilaria* sp. is a red seaweed, abundant in coastal areas of Bangladesh and a rich source of alkaloids, terpenoids, steroids, flavonoids, and phenolic compounds (Insani et al. 2022). Various phenolic compounds such as vanillic acid, gallic acid, cinnamic acid, p‐coumaric acid, hydroxybenzoic acid, phloroglucinol, and catechol are found in the *Gracilaria extracts that show* antioxidant activity and prevent cardiovascular complications (Sumayya et al. 2020). Sasadara and Wirawan (2021) stated that the ethanolic extracts of *Gracilaria* sp. had higher phenolic and flavonoid contents and showed effective radical scavenging activity. Generally, these bioactive compounds can inhibit the oxidation process in fish by donating electrons to free radicals, which prevents the generation of ROS (Hidayati et al. 2023). Moreover, bioactive compounds had notable bactericidal effects against various gram‐positive and gram‐negative fish spoilage bacteria (Husni and Wijaya 2013). Ethanoic extracts of *Gracilaria* sp. have also shown antibacterial activities and are especially effective against foodborne pathogens such as *Aeromonas hydrophila* , *Escherichia coli* , *Staphylococcus aureus* , and *Salmonella* sp. (Afonso et al. 2021; Mahendran et al. 2021). Deepitha et al. (2021) reported that the seaweed (*Padina tetrastromatica*) aqueous extracts enhance the shelf life of pangas fillets during chilled storage. Similarly, Shahrier et al. (2023) explored that the ethanolic extracts (2%, w/v) of *P. tetrastromatica*, *Sargassum natans* , and *S. fluitans* enhance the shelf life of refrigerated tilapia fillets. Besides, the storability of refrigerated red tilapia fillets increased when the fillets were dipped into *Gracilaria* sp. extracts (2%, w/v) (Husni and Wijaya 2013). However, published literature shows that no research has been performed yet regarding the effects of *Gracilaria* sp. extracts on the preservation of pangas fillets. Thus, this study intended to investigate the preservative effects of *Gracilaria* sp. extracts on the quality and shelf life of pangas fillets during refrigerated storage.

## 2 Methodology

### 2.1 Materials and Chemicals

The *Gracilaria* sp. was obtained from Saint Martin's Island, Cox's Bazar, Bangladesh. The seaweed was identified considering the botanical characteristics and brought to the laboratory. Then the seaweed was rinsed with clean water and kept at room temperature (27°C–29°C) for 7 days. The dried seaweed was ground into powder with a mixer grinder and kept at −26°C in zip bags (Shahrier et al. 2023). Live pangas ( *P. hypophthalmus* ) (average weight 2438 ± 95 g) were procured from a fish farm located in the Gazipur area of Bangladesh. The fish were killed immediately by hypothermia (ice‐cold water) and transferred to the Fish Processing Laboratory of Gazipur Agricultural University (GAU) in a heat‐resistant box with an ice‐to‐fish ratio of 2:1 (Kundu et al. 2024). The chemicals such as thiobarbituric acid (TBA), 1,1,3,3‐ tetraethoxypropane were procured from Fujifilm Wako Pure Chemical Corporation (Osaka, Japan). In this investigation, the bacteriological culture media (plate count agar, violet‐red bile agar, and mannitol salt agar) were bought from HiMedia, Mumbai, India. All other chemicals/reagents were of HPLC or analytical grade.

### 2.2 Preparation of *Gracilaria* sp. Extracts

The powdered seaweed (100 g) was extracted with ethanol (1000 mL, purity > 99.8%) (Shahrier et al. 2023). The extraction was done in an orbital shaker at 200 rpm for 8 h (Chakma et al. 2023). The liquid phase was filtered using Whatman No. 1 filter paper. The remnant was extracted again, filtered, and combined with the previously collected extracts. Then the extract was dried using a rotary evaporator under reduced pressure and weighed to ascertain the extract yield. The extracts were then kept at 4°C (dark conditions) for further use.

### 2.3 Preparation of Fish Fillets and Dipping Into *Gracilaria* sp. Extracts

The fish was immediately eviscerated and cleaned with potable water before being filleted into two parts from each fish. The fillets were sectioned into small pieces with an average weight of each piece of 50.65 ± 5.2 g. After that, the fillets were arbitrarily assigned into four treatment groups, and the first was the untreated control group (distilled water). The treatment groups were prepared by dipping the fillets into 1% (w/v), 2% (w/v), and 3% (w/v) of *Gracilaria* sp. extracts (GE) solution at 4°C for 10 min (Kundu et al. 2024). Then, the fillets were kept at ambient temperature for 5 min to develop an edible coating, packed in airtight polythene zip bags, and stored at 4°C ± 1°C in a refrigerator. The fillets from each group were randomly withdrawn for periodical (3 days) analysis until the spoilage of fish fillets.

### 2.4 Chemical Analysis

#### 2.4.1 Determination of Proximate Composition

The chemical composition such as crude protein, crude fat, ash, and moisture content of pangas fillets was evaluated by standard method given in Association of Official Analytical Chemists (AOAC 2006).

#### 2.4.2 Determination of pH

The pH was determined using the protocol outlined by Dulal et al. (2023). In summary, 10 g of fish sample was ground and mixed with distilled water (10 mL), and the mixture was filtered by Whatman No. 1 filter paper (GE Healthcare, Maidstone, UK). The pH was recorded in a pH meter (Jenway 3510, Staffordshire, UK).

#### 2.4.3 Determination of Peroxide Value (PV)

To determine the PV, the protocol proposed by Bligh and Dyer (1959) was used to extract the total lipid of fish fillets. The peroxide value of the extracted lipid was evaluated through the AOAC (2006) method and presented as meq O_2_/kg of lipid.

#### 2.4.4 Determination of Free Fatty Acid (FFA)

The FFA value of the fish fillets was estimated using the method proposed by Kirk and Sawer (1991). Fish fillet (10 g) was added to a mixture of anhydrous sodium sulfate (20 g) and chloroform (20 mL). The mixture was homogenized by a tissue homogenizer (Witeg Labortechnik GmbH, HG‐15A, Wertheim, Germany), and then filtered with Whatman No. 1 filter paper. Ethanol (10 mL) was added to it and titrated with 0.01 N NaOH. Phenolphthalein was used as an indicator to identify the end point that persisted for 15 s. The FFA was determined in triplicates, and the FFA content was stated as % oleic acid equivalent.

#### 2.4.5 Determination of Thiobarbituric Acid Reactive Substances

The TBARS was quantified following the method proposed by Buege and Aust (1978). Fish fillet (0.5 g) was blended with 2.5 mL of TBA solution, and the mixture was kept in a water bath at 95°C for 10 min. The solution was cooled and centrifuged at 3600 × *g* at ambient temperature for 20 min. The obtained supernatant was monitored at 532 nm using a UV/VIS spectrophotometer. A calibration curve was made using 1,1,3,3‐tetraethoxypropane at 0–10 ppm, and the value was presented as mg malondialdehyde (MDA)/kg fish flesh.

#### 2.4.6 Determination of Total Volatile Basic‐Nitrogen

The TVB‐N value was measured according to AOAC (2006). Shortly, 10 g of fish fillet was homogenized with cold 6% perchloric (90 mL) acid by a tissue homogenizer. After filtering the homogenate, the final volume of the filtrate was made up to 100 mL. The filtrate was loaded into the distillation unit and the distillate was collected in boric acid solution. The distillate was titrated with 0.01 M HCl. A mixed indicator was used to determine the end point, and the value was stated as mg nitrogen (N)/100 g of fish flesh.

### 2.5 Bacteriological Analysis

The changes in bacterial loads of fish fillets were measured according to the procedure of Kundu et al. (2024). Initially, 25 g of fish muscle was mixed with 0.85% sterile saline solution (225 mL) in a stomacher bag. After homogenization by a stomacher, the mixture was used to prepare decimal dilutions for bacteriological experiments. Plate count agar was used to detect aerobic plate count (APC) and psychrophilic bacteria count (PBC). For APC growth, plates were cultured at 37°C for 24 h, and at 7°C for 10 days in the case of PBC. The growth of Enterobacteriaceae was confirmed by violet‐red bile agar for 2 days at 37°C. Mannitol salt agar was used to culture *Staphylococcus* at 30°C for 2 days. All the values were stated as log_10_ colony forming units (CFU)/g of flesh.

### 2.6 Sensory Assessment

The fish fillets were evaluated by 10 experienced assessors of different ages from the Department of Fisheries Technology, GAU to test the sensory acceptability. The sensory team also followed a nine‐point hedonic scale constructed by Peryam and Pilgrim (1957) for the determination of odor, color, texture, and overall acceptability of the fish fillets. The sensory score of more than 5 was considered an acceptable threshold score. Sensory evaluation was conducted in separate sensory booths (1 × 1 m). The panelists evaluated the sensory attributes under controlled environmental conditions of temperature (24°C ± 1°C), relative humidity (around 50%), light, and other necessary parameters. Each sample was evaluated twice every 30 min interval between assessments.

### 2.7 Statistical Analyses

The experiments were set with triplicates for each parameter, and the values were denoted as mean ± SD. A two‐way ANOVA followed by a post hoc test was used to judge the significant variation among the means at a level of *p* < 0.05. All analyses were run with Statistix 10 (Analytical Software, Tallahassee, FL, USA). The multivariate analysis and Pearson's correlation were conducted using a ggplot2‐based R (R version 4.3.0, RStudio 2023.03.1 + 446) package named factoextra (http://www.sthda.com/english/rpkgs/factoextra), and FactoMineR (http://factominer.free.fr/index.html).

## 3 Results and Discussion

### 3.1 Proximate Composition

The proximate analysis showed that the pangas fillets contained 17.06% ± 0.25% crude protein and 4.43% ± 0.11% crude lipids, which indicates this fish is nutritious for human consumption. The moisture and ash contents of pangas fillets were 77.13% ± 0.17% and 1.30% ± 0.05%, respectively. Mansur et al. (2022) observed 73% moisture, 18% crude protein, 4.25% crude lipid, and 4.3% ash contents in pangas fillets. In another study, Deepitha et al. (2021) found 75.79% moisture, 18.09% crude protein, 2.55% crude lipid, and 0.89% ash contents in the fresh pangas. The results showed slight differences in the proximate percentages, and this is because of various factors such as fish size, gender, feeding habit, habitat, season of harvesting, and many environmental parameters (Rasul et al. 2021).

### 3.2 pH Value

**FIGURE 1:**
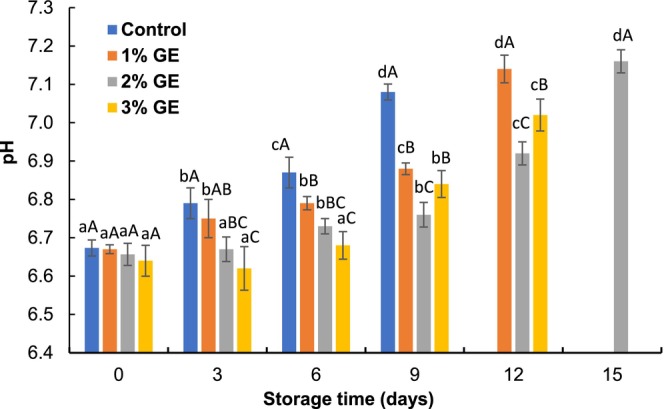
Changes in pH value of *P. hypophthalmus* fillets under refrigerated conditions. Lowercase letters of each line denote significant variations between the storage period within the same treatment, and uppercase letters denote significant variations among the treatments on the same preservation period at *p* < 0.05. The data denotes mean ± SD (*n* = 3).

Initially, the pH value of control and GE‐treated fillets ranged from 6.64 to 6.67 (Figure 1). The value of pH raised significantly (*p* < 0.05) during preservation, and the pH of control fillets exceeds the permitted range after 6th days of storage. The elevation of pH value could be related to the production of nitrogenous or volatile compounds and also the rapid degradation of nitrogenous compounds by bacterial activity (Jiménez‐Ruíz et al. 2023). In contrast, the lowest pH was observed in 2% GE‐treated fillets followed by 3% and 1% GE‐treated fillets on the 12th day of storage, and the values were 6.92, 7.14, and 7.02, respectively. A comparatively lower pH value found in the GE‐treated fillets could be the occurrence of several aromatic compounds that have antimicrobial activity and can inhibit the accretion of basic compounds (Devi and Kumari 2022; Sobuj et al. 2021). The findings of this experiment are in agreement with Shahrier et al. (2023), who noted that the pH was lower in tilapia fillets dipped into seaweed extracts than control at 4°C. Kundu et al. (2024) described that the 2% stevia leaf extract (SLE)‐treated fillets exhibited a slower increment of pH than the control fillets while stored at 4°C ± 1°C.

### 3.3 Total Volatile Basic‐Nitrogen (TBV‐N)

**FIGURE 2:**
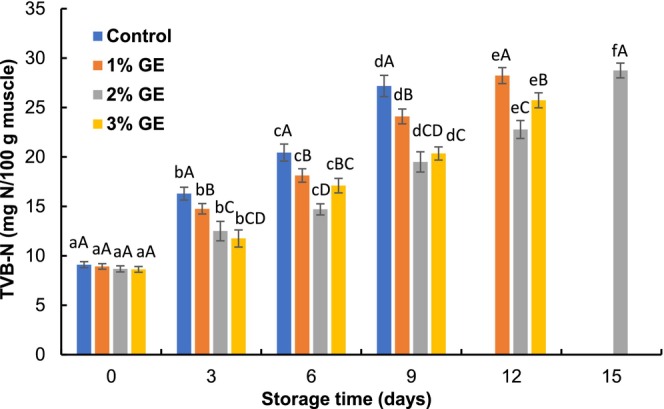
Changes in total volatile basic‐nitrogen (TVB‐N) values of *P. hypophthalmus* fillets under refrigerated conditions. Lowercase letters of each line denote significant variations between the storage period within the same treatment, and uppercase letters denote significant variations among the treatments on the same preservation period at *p* < 0.05. The data denotes mean ± SD (*n* = 3).

The TVB‐N values of control and GE‐treated fillets varied from 8.32 to 9.20 mg N/100 g of flesh at zero day (Figure 2). The TVB‐N of pangas fillets progressed significantly (*p* < 0.05) with the passage of time, and the values of control, 1%, 2%, and 3% GE‐treated fillets were 27.18, 28.23, 28.75, and 25.73 mg N/100 g at the 9th, 12th, 15th, and 12th days of storage, respectively. However, the GE‐treated fillets had a lower increment rate of TVB‐N formation, which reveals that GE can inhibit the bacterial activity for oxidative deaminization of NPN molecules (Kundu et al. 2024). Furthermore, the lower bacterial growth in the GE‐treated fillets reduced TVB‐N values, and this could be credited to the antibacterial compounds (mostly polyphenols) of the GE (Mahendran et al. 2021). Shahrier et al. (2023) reported that *Padina tetrastromatica* extract effectively retarded the TVB‐N accumulation in Nile tilapia fillets under refrigerated conditions. In this study, 2% GE‐treated fillets had an acceptable TVB‐N value (22.78 mg N/100 g), whereas 1% (28.23 mg N/100 g) and 3% GE‐treated fillets (25.73 mg N/100 g) crossed the permitted limit (< 25 mg N/100 g; Brijesh et al. 2009) at the 12th day of refrigerated condition. The higher TVB‐N values in the 3% GE‐treated fillets might be due to the higher enzymatic activity in the fillets, and similar findings were also observed by Kundu et al. (2024). They also explained that the higher phenolic compounds influenced the autooxidation process of fish and generated higher non‐protein nitrogenous compounds.

### 3.4 Free Fatty Acid (FFA)

**FIGURE 3:**
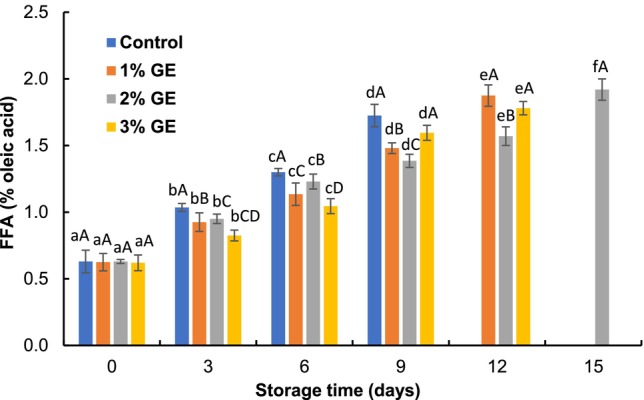
Changes in free fatty acid (FFA) values of *P. hypophthalmus* fillets under refrigerated conditions. Lowercase letters of each line denote significant variations between the storage period within the same treatment, and uppercase letters denote significant variations among the treatments on the same preservation period at *p* < 0.05. The data denotes mean ± SD (*n* = 3).

The FFA of the control pangas fillet was 0.63% oleic acid and the value reached 1.73% oleic acid on the 9th day of refrigerated storage (Figure 3). The GE‐treated fillets displayed significantly (*p* < 0.05) lower FFA values than the control fillets, and the values reached 0.63 to 1.88% for 1% GE, 0.63 to 1.92% for 2% GE, and 0.62 to 1.78% oleic acid for 3% GE‐treated fillets at the 12th, 15th, and 12th days of refrigerated preservation, respectively. The results exhibited that the increment rate of FFA values was higher in the control fillets than in the GE‐treated fillets under refrigerated conditions. Comparatively lower amounts of FFA in the GE‐treated fillets might be due to GE containing bioactive compounds (especially phenolics), which inhibit the formation of FFA in the fish fillets (Insani et al. 2022). Furthermore, 2% GE‐treated fillets showed a slower FFA increment rate than 3% GE‐treated fillets, and this could be due to 2% GE inhibiting the formation of free radicals and interacting with the reactive oxygen species to delay the self‐oxidation process (Ibrahim et al. 2022). The outcome of this study is similar to the findings of Kundu et al. (2024), who observed that 2% SLE treated fillets significantly (*p* < 0.05) hindered the formation of FFA compared to 3% SLE‐treated fillets under refrigerated conditions. Similarly, Khadem et al. (2020) found that the *Capparis spinosa* extracts (0.5%) were more effective in reducing the hydrolysis of lipids of rainbow trout fillets when stored at 4°C.

### 3.5 Peroxide Value (PV)

**FIGURE 4:**
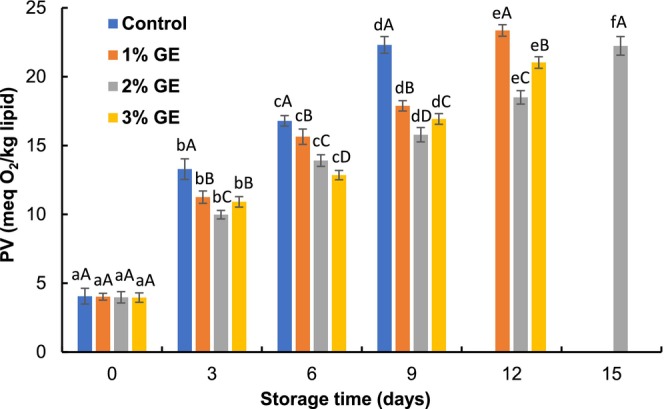
Changes in peroxide values (PV) of *P. hypophthalmus* fillets under refrigerated conditions. Lowercase letters of each line denote significant variations between the storage period within the same treatment, and uppercase letters denote significant variations among the treatments on the same preservation period at *p* < 0.05. The data denotes mean ± SD (*n* = 3).

The PV of pangas fillets at zero day varied from 3.95 to 4.05 meq O_2_/kg lipid (Figure 4). The PV of control, 1%, 2%, and 3% GE‐treated fillets was 22.31, 23.35, 22.24, and 21.03 meq O_2_/kg lipid at 9th, 12th, 15th, and 12th days of preservation, respectively. The PV of control fillets exceeded the acceptable limit (PV < 20 meq O_2_/kg lipid; Kundu et al. 2024) after 6th day of storage. However, the progression rate of PV was remarkably higher in 1% and 3% GE‐treated fillets compared to 2% GE‐treated fillets (*p* < 0.05). The higher PV in the 1% and 3% GE‐treated fillets might be due to the higher lipid peroxidation, while 2% GE‐treated fillets were more effective in lowering PV during refrigerated conditions. The effectiveness of the GE‐treated fillets suggests that GE contains several polyphenolic compounds (such as gallic acid, caffeic acid, quercetin, luteolin, kaempferol), which inhibit the formation of peroxides in lipids (Mahendran et al. 2021; Ouahabi et al. 2023). A related finding was found by Takyar et al. (2019), who reported that *Spirulina platensis* and *Chlorella vulgaris* extracts (0.1%) were effective in reducing peroxide formation during refrigerated storage of rainbow trout. John and Siddappaji (2019) also described that the PV of tea extract‐treated mackerel fillets showed a lower value than control fillets during icing.

### 3.6 Thiobarbituric Acid Reactive Substances (TBARS)

**FIGURE 5:**
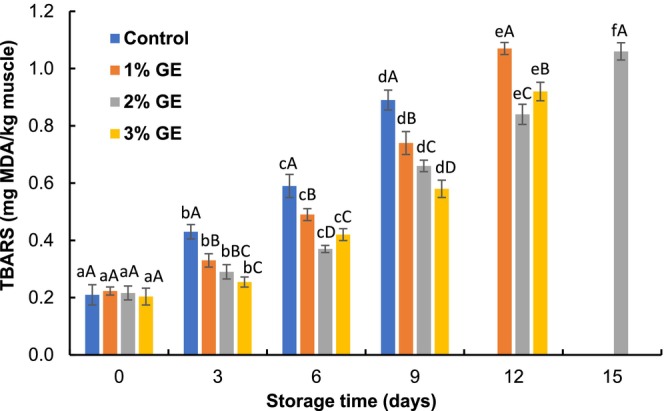
Changes in thiobarbituric acid reactive substances (TBARS) of *P. hypophthalmus* fillets under refrigerated conditions. Lowercase letters of each line denote significant variations between the storage period within the same treatment, and uppercase letters denote significant variations among the treatments on the same preservation period at *p* < 0.05. The data denotes mean ± SD (*n* = 3).

The initial TBARS values of pangas fillets varied from 0.20 to 0.22 mg MDA/kg fish muscle (Figure 5). The TBARS of control, 1%, 2%, and 3% GE‐treated fillets increased significantly (*p* < 0.05) during the preservation period and reached 0.89, 1.07, 1.06, and 0.92 mg MDA/kg at the 9th, 12th, 15th, and 12th days of preservation, respectively. The TBARS increment rate was lower in the GE‐treated fillets than control, and the 2% GE‐treated fillets showed the lowest value. The lower progress rate of TBARS generation in GE‐treated fillets indicates that GE effectively reduced the MDA production in pangas fish fillets (Afrin et al. 2023). This result is comparable with Shahrier et al. (2023), who noted that *P. tetrastromatica* extracts reduced the increment of TBARS in tilapia fillets when stored at 4°C. Deepitha et al. (2021) also reported that *P. tetrastromatica* extract (0.5% and 1%) reduced the TBARS in pangas fillets during chilled conditions. Moreover, 1% aqueous rosemary extract significantly reduced the TBA value in *Seriola dumeriri* fillets kept at 4°C compared to control (Ibrahim et al. 2022).

### 3.7 Bacteriological Analyses

**FIGURE 6:**
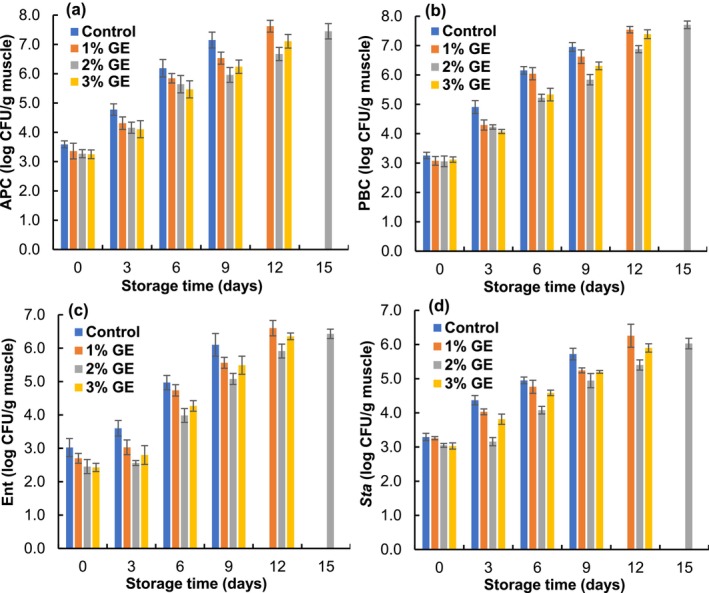
Changes in (a) aerobic plate count, (b) psychrophilic bacterial count, (c) Enterobacteriaceae count, and (d) *Staphylococcus* count of *P. hypophthalmus* fillets under refrigerated condition. The data denotes means ± SD (*n* = 3). APC, aerobic plate count; Ent, Enterobacteriaceae; GE, *Gracilaria* sp. extracts; PBC, psychrophilic bacterial count; Sta, Staphylococcus.

The changes in bacterial count of pangas fillets are expressed in Figure 6. The APC ranged from 3.25 to 3.59 log CFU/g at zero day for all the treatments, and the values gradually increased (*p* < 0.05) over the storage period. The APC of the GE‐treated and control fillets crossed the acceptable limit (7 log CFU/g muscle for fresh fish; ICMSF 1986) on the 9th day for control (7.15 log CFU/g), 12th day for 1% (7.62 log CFU/g), and 3% (7.11 log CFU/g), and 15th day for 2% (7.45 log CFU/g) GE‐treated fillets, respectively. Results showed that the GE‐treated fillets exhibited a lower bacterial count than the control fillets, and 2% GE‐treated fillets showed the lowest value during refrigerated storage. These results explain that phenolic compounds in GE disrupt the bacterial cell wall by their benzene ring structure (Insani et al. 2022). Moreover, the hydroxyl group of phenolic compounds has easily penetrated into the bacterial cell and bonded with enzymes, which causes cell degeneration (Deepitha et al. 2021). This result matches with Ibrahim et al. (2022), who found that 1% rosemary extracts contributed to a notable decline in the total bacterial count of *S. dumerili* fillet when stored at 2°C.

The PBC ranged between 3.06 and 3.26 log CFU/g at zero day (Figure 6). The PBC was moderately increased in all the treatments under refrigerated storage (*p* < 0.05). The PBC for control was 6.95 on the 9th day, 7.54 for 1% on the 12th day, 7.71 for 2% on the 15th day, and 7.39 log CFU/g for 3% GE‐treated fillets on the 12th day of storage. However, the PBC was notably lower in GE‐treated fillets than in control fillets, which reveals that GE contains various phenolic compounds that exhibit antimicrobial activity (Ouahabi et al. 2023). The bacterial cell envelope synthesis and enzymatic activity are halted when the bacteria come closer to the polyphenolic compounds of GE, and therefore, the counts remain lower in the GE‐treated fillets than in control (Silva et al. 2020). Shahrier et al. (2023) also stated that seaweed (*S. natans, S. fluitans*, and *P. tetrastromatica*) extracts solution (2%, w/v) significantly (*p* < 0.05) reduced the PBC count of Nile tilapia fillets under refrigerated conditions for 20 days.

The initial Enterobacteriaceae count varied between 2.43 and 3.02 log CFU/g (Figure 6). Results showed that the bacterial load was gradually (*p* < 0.05) increased in all the treatments, and the Enterobacteriaceae count was reasonably slower in the GE‐treated fillets than control fillets during storage. The values reached 6.10, 6.60, 6.43, and 6.35 log CFU/g for control, 1%, 2%, and 3% GE‐treated fillets at the 9th, 12th, 15th and 12th days of storage, respectively. However, the lower values in the GE‐treated fillets might be due to the antimicrobial activities of various bioactive constituents, especially alkaloids, polyphenols, tannins, and catechin in the GE (Silva et al. 2020). Moreover, Assaw et al. (2018) observed that *Gracilaria* sp. extracts showed significant efficacy against *Escherichia coli* and many other bacteria. The findings are in agreement with Kundu et al. (2024), who described that the SLE extract solution (2%, w/v) significantly (*p* < 0.05) hindered the Enterobacteriaceae count in tilapia fillets at 4°C ± 1°C.

The initial *Staphylococcus* count in pangas fillets ranged from 3.03 to 3.29 log CFU/g, and the counts were raised (*p* < 0.05) gradually during the storage time (Figure 6). The maximal *Staphylococcus* count was 5.72, 6.26, 6.03, and 5.90 log CFU/g at the 9th, 12th, 15th and 12th days for control, 1%, 2%, and 3% GE‐treated fillets, respectively, and each value remained within the allowable limit. However, the advancement rate of *Staphylococcus* count was moderately slower in GE‐treated fillets that might be due to the antibacterial properties of GE (Afonso et al. 2021). Among the treatments, 2% GE‐treated fillets had the lowest count compared to other treatments. An indistinguishable outcome was found by Kundu et al. (2024), who confirmed that the *Staphylococcus* count of 2% SLE‐treated fillets was relatively below the levels found in other fillets.

### 3.8 Sensory Assessment

The fillets were evaluated based on a 9‐point hedonic scale, and a score of 5 or more was considered satisfactory. The sensory characteristics of pangas fillets are presented in Table 1. Results showed that the sensory characteristics such as overall acceptability, odor, color, and texture of the control, 1%, 2%, and 3% GE‐treated fillets were consumable up to the 6th, 9th, 12th, and 9th days of preservation, respectively. The sensory score of all the treatments was decreased during preservation (*p* < 0.05). However, the 2% GE‐treated fillets demonstrated better sensory quality followed by the 3%, 1% GE‐treated, and control fillets, which might be due to the preservative effects of phenolic molecules of GE (Insani et al. 2022). The chemical and bacterial analysis data also support the sensory evaluation results, and this phenomenon is relevant to the results of Shahrier et al. (2023). Moreover, our results are consistent with the outcome of Deepitha et al. (2021), who found that *P*. *tetratromatica* extract (2%) showed the highest sensory value of pangas fillets compared to other treatments during chilled conditions.

**TABLE 1:** Changes in sensory attributes of pangas fillets under refrigerated condition.

Treatments	Storage time (days)					
	0	3	6	9	12	15
**Color**						
Control	8.86 ± 0.06^dA^	8.02 ± 0.34^cB^	7.15 ± 0.22^bC^	4.81 ± 0.11^aD^		
1% GE	8.83 ± 0.09^eA^	8.13 ± 0.43^dAB^	7.76 ± 0.16^cB^	6.74 ± 0.17^bC^	4.29 ± 0.24^aC^	
2% GE	8.81 ± 0.05^fA^	8.43 ± 0.19^eA^	8.08 ± 0.15^dA^	7.52 ± 0.24^cA^	6.92 ± 0.15^bA^	4.85 ± 0.14^aA^
3% GE	8.84 ± 0.06^eA^	8.27 ± 0.26^dAB^	7.99 ± 0.17^cA^	7.10 ± 0.20^bB^	4.79 ± 0.28^aB^	
**Odor**						
Control	8.89 ± 0.06^dA^	7.88 ± 0.20^cC^	6.52 ± 0.26^bD^	3.95 ± 0.22^aD^		
1% GE	8.87 ± 0.03^eA^	7.98 ± 0.26^dBC^	7.46 ± 0.19^cC^	6.87 ± 0.14^bC^	4.12 ± 0.23^aC^	
2% GE	8.91 ± 0.04^fA^	8.33 ± 0.23^eA^	8.02 ± 0.24^dA^	7.43 ± 0.18^cA^	6.47 ± 0.17^bA^	4.33 ± 0.28^aA^
3% GE	8.90 ± 0.02^eA^	8.20 ± 0.21^dAB^	7.73 ± 0.25^cB^	7.14 ± 0.10^bB^	4.45 ± 0.15^aB^	
**Texture**						
Control	8.91 ± 0.04d^AB^	8.12 ± 0.24^cB^	6.71 ± 0.23^bC^	4.07 ± 0.22^aC^		
1% GE	8.92 ± 0.03e^AB^	8.24 ± 0.30^dB^	7.53 ± 0.25^cB^	6.40 ± 0.18^bB^	4.35 ± 0.19^aB^	
2% GE	8.94 ± 0.02^fA^	8.57 ± 0.18^eA^	8.13 ± 0.29^dA^	7.67 ± 0.15^cA^	6.56 ± 0.47^bA^	4.47 ± 0.21^aA^
3% GE	8.90 ± 0.02^eB^	8.38 ± 0.22d^AB^	7.81 ± 0.34^cB^	7.53 ± 0.25^bA^	4.64 ± 0.18^aB^	
**Overall acceptability**						
Control	8.89 ± 0.04^dA^	8.01 ± 0.12^cB^	6.80 ± 0.32^bC^	4.28 ± 0.29^aD^		
1% GE	8.87 ± 0.03^eA^	8.11 ± 0.15^dB^	7.58 ± 0.28^cB^	6.67 ± 0.21^bC^	4.26 ± 0.46^aB^	
2% GE	8.88 ± 0.03^fA^	8.44 ± 0.16^eA^	8.08 ± 0.19^dA^	7.54 ± 0.18^cA^	6.65 ± 0.65^bA^	4.55 ± 0.16^aA^
3% GE	8.88 ± 0.02^eA^	8.28 ± 0.28^dA^	7.84 ± 0.24^cA^	7.25 ± 0.43^bB^	4.63 ± 0.31^aB^	

*Note:* Values are expressed as means ± SD (*n* = 7). Different uppercase letters designate significant (*p* < 0.05) variations among various treatments on the same storage time, while the lowercase letters designate significant (*p* < 0.05) variations among various storage periods on the same treatment. The satisfactory sensory score is above 5.0 for raw fish.

Abbreviation: GE, *Gracilaria* sp. extracts.

### 3.9 Correlation Among Various Chemical, Bacteriological, and Sensory Characteristics

**FIGURE 7:**
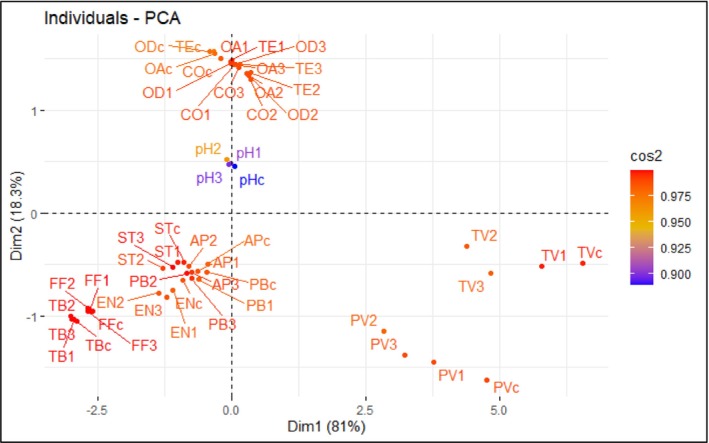
PCA score plot of chemical, bacteriological, and sensory attributes of *P. hypophthalmus* fillets during preservation period. AP, aerobic plate count; c, control; CO, color; EN, Enterobacteriaceae; FF, free fatty acid; OA, overall acceptability; OD, odor; PB, psychrophilic bacteria; PV, peroxide value; ST, *Staphylococcus*; TB, thiobarbituric acid reactive substances; TE, texture; TV, total volatile basic‐nitrogen; 1 = 1% GE, 2 = 2% GE, 3 = 3% GE.

Principal component analysis (PCA) was conducted to explore the relationships between the chemical (pH, TVB‐N, FFA, PV, TBARS), bacterial (PBC, APC, *Staphylococcus*, and Enterobacteriaceae), and sensory characteristics of pangas fillets stored under refrigerated conditions. The PC1 and PC2 accounted for 81% and 18.3% of the total variation in the dataset, respectively (Figure 7). In PC1, PV and TVB‐N values are well separated from other groups, which indicates their distinct characteristics and had a substantial effect on quality characteristics. The sensory parameters and bacteriological parameters of the treated and control fillets showed another cluster of similarity and strongly contributed to PC1 and PC2. However, PC2 defined an inverse relationship between sensory characteristics and microbiological counts of fish fillets. A positive linear relationship was found between bacterial and chemical parameters (*r* = 0.72–0.99), whereas an inverse relationship was observed between chemical and sensory characteristics (*r* = −0.73 to −0.99), and microbiological and sensory characteristics (*r* = −0.84 to −0.99) of fish fillets throughout the refrigerated time (Figure 7). An indistinguishable result was also reported by Kundu et al. (2024).

## 4 Conclusion

Chemical deterioration and rapid growth of bacteria are responsible for physiological changes and organoleptic alterations in pangas fillets under refrigerated conditions. In this study, *Gracilaria* sp. extract was used as a natural antioxidant and antibacterial agent, which positively impacted the quality attributes of pangas fillets. The accumulation of lipid oxidation products leads to rapid deterioration of the fillet's quality, where 2% GE‐treated fillets showed the lowest TBARS value; however, the exact amount of primary and secondary lipid degradation compounds is unknown. In addition, the total volatile basic nitrogen value effectively decreased in the 2% GE‐treated fillets, and the fillets were consumable up to the 12th day of storage with little change in sensory qualities. Moreover, 2% GE‐treated fillets significantly retarded the growth of different fish spoilage bacteria and enhanced the shelf life by 6 days more than the untreated fillets at 4°C ± 1°C. Therefore, *Gracilaria* sp. extract can be used as a natural additive in pangas fillet preservation at the industrial level to extend the shelf life. However, further studies are needed to obtain a more precise effect of this extract on lipid oxidation products.

## Author Contributions

**Md Apon Dulal:** conceptualization (equal), data curation (lead), funding acquisition (lead), investigation (equal), methodology (equal), project administration (lead), resources (lead), writing – original draft (lead). **Rokshana Islam:** data curation (equal), investigation (equal), methodology (equal), writing – review and editing (equal). **Shoumik Ahmed:** data curation (equal), investigation (equal), methodology (equal), writing – review and editing (equal). **Chunhong Yuan:** data curation (equal), formal analysis (lead), writing – review and editing (equal). **A K M Azad Shah:** conceptualization (equal), supervision (lead), validation (lead), writing – review and editing (equal).

## Ethics Statement

This study was conducted following the guidelines established by the research ethics committee, GAU, Bangladesh (Ref. No. FVMAS/AREC/2023/52).

## Conflicts of Interest

The authors declare no conflicts of interest.

## Data Availability

Data will be made available upon request.
